# Primary Proximal femur replacement for unstable osteoporotic intertrochanteric and subtrochanteric fractures in the elderly: A retrospective case series

**DOI:** 10.1016/j.amsu.2019.07.014

**Published:** 2019-07-11

**Authors:** Shah Fahad, Muhammed Zohaib Nawaz Khan, Mujahid Jamil Khattak, Masood Umer, Pervaiz Hashmi

**Affiliations:** Department of Surgery, AKUH, Pakistan

**Keywords:** Proximal femur replacement, Intertrochanteric fracture, Dislocation, Mortality

## Abstract

**Background:**

Hip fractures usually occur in old aged patients with osteoporotic bone. Management of hip fractures in old aged patient is aimed to ambulate patient immediately and to restore the pre-operative ambulation. Proximal femur replacement is an effective treatment option in elderly patient with osteoporotic bones. It allow immediate weight bearing and early return to preoperative ambulatory status and minimizes the chances of systemic complication associated with prolong bed rest associated with internal fixation. This study is aimed to review the outcome of the patients whom underwent proximal femur replacement as primary treatment for the patient with comminuted intertrochanteric and sub trochanteric fracture.

**Patients and method:**

This is a study conducted in our university hospital which is a tertiary-care level-1 trauma center. A retrospective analysis of 21 patients who underwent proximal femur replacement for comminuted intertrochanteric and sub trochanteric fracture, age more than 60 years during the period from April 2011 to March 2018 was conducted. Data collected included: age, gender, comorbidities, mechanism of injury, type of fracture, functional outcome (calculated via Harris Hip Score) and one year mortality.

**Results:**

The mean age of the patients was 74.05(range 64–91) years, out of which 13 (61.8%) were female and 8 (38.0%) were male. The mean follow up was 32.6(8–91 months).Immediate post-operative ambulation status was full weight bearing (FWB) in 17 patients (80.9%) of the patients while three patients (19.0%) had non-weight bearing (NWB) due to associated co-morbidities. The mean preoperative Harris Hip score was 68.0, while the mean postoperative Harris Hip score was 66.5 at last follow up. Post operatively one patient (4.7%) developed pulmonary embolism, one patient developed dislocation. One patent (4.7%) died of sepsis from implant infection at 8 months after surgery

**Conclusion:**

Primary Proximal femoral replacement in a viable option in old aged patients with poor bone quality who developed intertrochanteric and subtrochanteric fracture. According to our study, with mortality rate comparable to that of primary fixation, yet with the added advantage of immediate post op ambulation and reduced incidences of decubitus ulcers, atelectasis and DVT.

## Highlights

• Management of hip fractures in old aged patient is aimed to ambulate patient immediately.
• Proximal femur replacement is an effective treatment option in elderly patient with osteoporotic bones.
• Proximal femur replacement allows immediate weight bearing and early return to preoperative ambulatory status.

## 1 Background

Hip fractures usually occur in old aged patients with osteoporotic bone. Management of hip fractures in these patients with osteoporotic, poor bone stock bones is a challenge. Internal fixation of these fractures usually results in failure and are associated with prolong period of bed rest and restricted ambulation which is further complicated by deep venous thrombosis and pulmonary embolism [1].

Management of hip fractures in old aged patient is aimed to ambulate patient immediately and to restore the pre-operative ambulation status as soon as possible to prevent systemic complication and improve survival [1].

Proximal femur replacement is an effective treatment option in elderly patient with osteoporotic bones and in patient with severe bone loss at proximal femur. It allows immediate weight bearing and early return to preoperative ambulatory status and eliminate the chances of avascular necrosis of femur head and minimizes the chances of systemic complication associated with prolong bed rest associated with internal fixation [2].

This study is aimed to review the outcome of the patients whom underwent proximal femur replacement as primary treatment for the patient with comminuted intertrochanteric and sub trochanteric fracture.

## 2 Materials and methods

This is a study conducted in our university hospital which is a tertiary-care level-1 trauma center. We obtained the hospital ethical review committee approval and registered the study in data registry.

A retrospective analysis of 21 patients who underwent proximal femur replacement during the period from April 2011 to March 2018 was conducted. All orthopaedic patients who underwent proximal femur replacement for comminuted intertrochanteric and sub trochanteric fracture, age more than 60 years, ambulatory patient before trauma with complete follow up for one year were enrolled into the study. Patients with missing records and those who were lost to follow-up, nonambulatory patients before injury, patients with pathological fractures and patient with associated other fractures were excluded. Data collected included: age, gender, comorbidities (Table 1), mechanism of injury, type of fracture and the mean operative time, functional outcome (calculated via Harris Hip Score) and one year mortality. The work has been reported in line with the PROCESS criteria [15].

**Table 1:** Comorbidities of the patients.

Comorbidities	Number	Percentage (%)
**Hypertension**	**13**	**61.9**
**Diabetes Mellitus**	**4**	**19.0**
**Ischemic heart disease**	**2**	**9.5**
**Asthma**	**2**	**9.5**
**COPD**	**1**	**4.7**
**CKD**	**2**	**9.5**
**Thalassemia Minor**	**2**	**9.5**

Pre-operative assessment included a thorough history, workup and physical examination. Medical co-morbidities were recorded and controllable risk factors identified and optimized before surgery. Radiographic evaluation included radiographs AP pelvis and femur anteroposterior and lateral shoot through. Laboratory investigations included complete blood count, serum urea, creatinine, electrolytes, blood sugar random and urine routine examination.

All procedures were performed under general anaesthesia and spinal anaesthesia. Intravenous prophylactic antibiotics were administered at the time of induction of anaesthesia as according to our institution's guidelines. All procedures were performed by fellowship trained consultants and as per surgeon preference through lateral or posterior approach. Preoperatively, templating was done using of fractured and contralateral hip plain radiographs to measure femoral head size, canal diameter as well length tip of greater trochanter to lesser trochanter. Careful exposure of fracture was done sparing abductor mechanism and already intact grater trochanter. After removing head and clearing acetabulum, femoral canal preparation was done by using progressive reamer sizes until adequate fit achieved. Same size stem as last reamer was used to trial with a body size to recreate original metaphyseal component of patient used, head and neck size adjusted to achieve ample stability with full functional range of motion, maintenance of tissue tension and limb length equality. Implant used included standard 170mm cementless HA coated femoral stems as well as HA coated body with tendon holes; bipolar head was size 28 or 32 as per cup size, stainless steel head with high molecular weight polyethylene. After implantation of the definitive prosthesis, heavy non-absorbable sutures were used to anchor the great trochanter and abductors to the lateral part of proximal region of the prosthesis dedicated holes are present. If abductors were available to be tagged to the prosthesis, an abduction brace was used for six weeks.

**Fig. 1:**
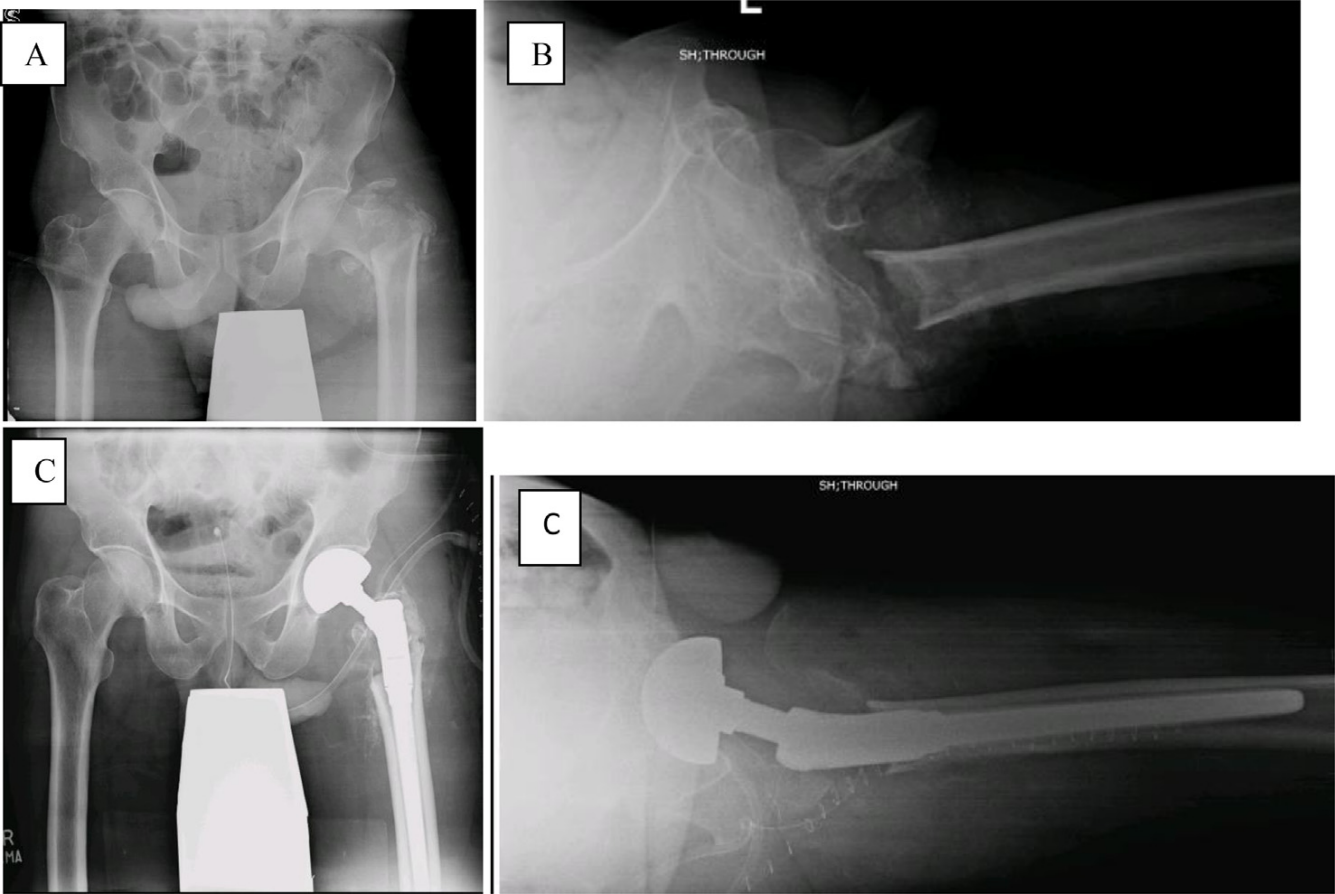
Pre-operative x-rays pelvis AP [A] and left hip lateral shoot through [B] Showing intertrochanteric fracture. Post-operative x-rays pelvis AP [C] and left hip lateral shoot through [D] showing proximal femur replacement.

Post-operatively, the patients were allowed weight-bearing with ambulation as tolerated with the help of walker. Quadriceps muscle strengthening exercises were started from one day after surgery. DVT prophylaxis was given as low molecular weight heparin to all patients. Patients were discharged after an average of three days and followed-up one week, two weeks and six monthly during first year after surgery and yearly subsequently. At every visit, the patients were examined clinically for wound healing, postoperative ambulation status, need for walking aid, and postoperative complications. Radiographs were evaluated for the evidence of loosening (Fig. 1). Functional assessment was done via Harris Hip Score.

## 3 Results

The mean age of the patients was 74.05(range 64–91) years, out of which 13 (61.8%) were female and 8 (38.0%) were male. The mean follow up was 32.6(8–91 months).Immediate post-operative ambulation status was full weight bearing (FWB) in 17 patients (80.9%) of the patients while three patients (19.0%) had non-weight bearing (NWB) due to associated co-morbidities. The mean preoperative Harris Hip score was 68.0, while the mean postoperative Harris Hip score was 66.5 at last follow up. Post operatively one patient developed pulmonary embolism which was managed with supportive care. One patient developed dislocation on first postoperative day which was managed with closed reduction. One patent died of sepsis from implant infection at 8 months after surgery.

## 4 Discussion

Although the use of hemiarthoplasty for unstable intertrochanteric fractures was first proposed in 1971, its use has been limited and controversial when compared to its popularity in neck of femur fractures. Due to the immense success of internal fixation in reducing mortality associated with intertrochanteric fractures, the need for any other modalities of treatment seemed unnecessary. Though there is questionable success when it comes to unstable fractures of the elderly with limited and poor quality bone stock. The rate of failure of dynamic hip screw is 6.8–9.8% while failure rate of proximal femur nail is 7.1–12.5% in patient with unstable intertrochanteric fractures with osteoporosis. The weak osteoporotic bone do not provide strong purchase for the screws which lead to mechanical failure, collapse and migration of femur head into varus and retroversion which leads to limb shortening and limping and cut out of the screw from the femur head leading to pain and functional disability [5,6].This mechanical failure is further compounded by medical complication caused by poor general health, medical comorbidities and poor ambulation after surgery [1].

Mortality in elderly patients after hip fracture is a major concern. Fracture in these patients are further compounded by their comorbidities, complication resulted from immobilization leading to enhance mortality [8,9]. Postoperative mortality is a concern after hip fracture surgery.A study by Dobbs RE et al., compared the 30 days mortality in patient with intertrochanteric fracture treated with internal fixation vs arthroplasty and didn't find any significant difference in mortality, 4.5% with internal fixation while 4.8% with arthroplasty [10]. In our series of 21 patients, one patient (4.7%) died within 12 months of surgery due to implant infection leading to sepsis.

With the initial works of Tronzo, Rosenfeld, Schwartz, and Alter reporting good results with the use of the prosthesis [12, 13], numerous other studies have found similar results. With definitive advantages such as early ambulation and earlier return to preinjury status, Stein and Goldstein proclaimed the same notion with the use of the Leinback prosthesis [14]. Furthermore, a reduction in complications, mortality rates, improvement in the patient's living quality, and reduction in the burden of the patient's family was noted by Liang et al. In our study, majority of patients were ambulated on the 1st post-operative day with a walker and support, complications such as pressure sores, DVT and atelectasis due to prolonged recumbency were unseen and Harris hip score was restored to pre injury levels. Early ambulation also allowed earlier discharge from hospital and theoretically lesser exposure to hospital acquired infections.

## 5 Conclusion

Despite a myriad of available options and advanced implants obtainable, hip fracture surgery still presents with significant morbidity and mortality. In stable fracture with good bone stock, internal fixation remains the gold standard for treatment, reducing mortality drastically [11]; though elderly patients with poor bone stock, comminuted/unstable fractures and poor screw fixation becomes an area of debate due to the significant number of mechanical complications [3] and implant failure [3,4]. Proximal femoral replacement in such scenarios presents a viable option, according to our study, with a mortality rate comparable to that of primary fixation, yet with the added advantage of immediate post op ambulation and reduced incidences of decubitus ulcers, atelectasis and DVT.

Limitation of the study: Retrospective design of the study, no comparative group, small sample size.

We recommend prospective randomize study with large sample size.

## Ethical approval

Hospital ethical review committee approval was taken.

## Sources of funding

None.

## Author contribution

Shah Fahad: first proposal, data collection, analysis and manuscript writing and editing.

Muhammad Zohaib Nawaz Khan: data analysis and manuscript writing and editing.

Mujahid Jamil: review and editing.

Pervaiz Hashmi: review and editing.

Masood Umer: final review.

## Conflicts of interest

No conflict of interest.

## Trial registry number

Registration of the study in data registry with research registry NCT03875027.

## Guarantor

Pervaiz Hashmi.

Shah Fahad.

## Provenance and peer review

Not commissioned, internally reviewed.
